# The role of EGFR mutation as a prognostic factor in survival after diagnosis of brain metastasis in non-small cell lung cancer: a systematic review and meta-analysis

**DOI:** 10.1186/s12885-019-5331-z

**Published:** 2019-02-13

**Authors:** Wen-Ya Li, Ting-Ting Zhao, Hui-Mian Xu, Zhen-Ning Wang, Ying-Ying Xu, Yunan Han, Yong-Xi Song, Jian-Hua Wu, Hao Xu, Song-Cheng Yin, Xing-Yu Liu, Zhi-Feng Miao

**Affiliations:** 1 Department of Thoracic Surgery, First Hospital of China Medical University, Shenyang, Liaoning Province China; 2 Department of Breast Surgery, First Hospital of China Medical University, Shenyang, Liaoning Province China; 3 Department of Surgical Oncology, First Hospital of China Medical University, Shenyang, 110001 Liaoning Province China; 4 Division of Public Health Sciences, Department of Surgery, Washington University School of Medicine, Saint Louis, MO USA; 5 Department of Medical Oncology, Shengjing Hospital of China Medical University, Shenyang, Liaoning Province China

**Keywords:** Lung cancer, EGFR, Brain metastasis, Prognosis, Meta analysis

## Abstract

**Background:**

The brain is a common site for metastasis in non-small-cell lung cancer (NSCLC). This study was designed to evaluate the relationship between the mutational of the epidermal growth factor receptor (EGFR) and overall survival (OS) in NSCLC patients with brain metastases.

**Methods:**

Searches were performed in PubMed, EmBase, and the Cochrane Library to identify studies evaluating the association of EGFR mutation with OS in NSCLC patients through September 2017.

**Results:**

4373 NSCLC patients with brain metastases in 18 studies were involved. Mutated EGFR associated with significantly improved OS compared with wild type. Subgroup analyses suggested that this relationship persisted in studies conducted in Eastern, with retrospective design, with sample size ≥500, mean age of patients ≥65.0 years, percentage male < 50.0%, percentage of patients receiving tyrosine kinase inhibitor ≥30.0%. Finally, although significant publication bias was observed using the Egger test, the results were not changed after adjustment using the trim and fill method.

**Conclusions:**

This meta-analysis suggests that EGFR mutation is an important predictive factor linked to improved OS for NSCLC patients with brain metastases. It can serve as a useful index in the prognostic assessment of NSCLC patients with brain metastases.

**Electronic supplementary material:**

The online version of this article (10.1186/s12885-019-5331-z) contains supplementary material, which is available to authorized users.

## Background

Lung cancer remains the leading cause of cancer-related deaths worldwide, and nearly 85% of lung patients are diagnosed with non-small-cell lung cancer (NSCLC) [1]. Despite continued efforts, innovations, and progress in diagnosis and treatment, the 5-year overall survival (OS) for patients with advanced NSCLC is only 15% [2]. Over 25% of NSCLC patients present with brain metastases at diagnosis, and nearly 45% of autopsies of NSCLC patients show brain metastasis [3–5]. The median OS of lung cancer patients with brain metastases with no treatment ranges from 4 to 11 weeks [5], while this can be prolonged to 14 months if they receive local treatments such as neurosurgery, stereotactic radiosurgery, or brain radiation therapy [6]. To best improve the OS and quality of life for patients receiving local treatments, selection of the optimal treatment option should be based on definite prognostic factors of each specific patient.

Genetic alterations of the epidermal growth factor receptor (EGFR) occur in approximately 20% of patients with lung adenocarcinomas in Western countries and 40–60% in East Asia for [7–11]. Currently, tyrosine kinase inhibitors (TKIs) are widely used in NSCLC according to the EGFR mutation type. TKIs can reduce the incidence of long-term treatment failure in patients with or without brain metastases, possibly through EGFR signaling pathways promoting factors associated with oncogenic and metastatic progression such as angiogenesis, cellular proliferation, and epithelial mesenchymal transition [12, 13]. Although these relationships have been demonstrated, the association of EGFR mutation with OS in NSCLC patients with brain metastases remains controversial.

Several studies have illustrated that EGFR mutations are significantly associated with longer OS in NSCLC patients with brain metastases [14–19], yet numerous other studies show no association between EGFR mutations and OS for these patients [20–29]. Two studies have even suggested that EGFR mutations may decrease OS [30, 31]. Clarifying the relationship between EGFR and OS in NSCLC patients with brain metastases is particularly important, as it has not been definitively determined. Therefore, we conducted a meta-analysis of available studies to determine if a correlation exists between EGFR status and OS in NSCLC patients with brain metastases. The relationship was further quantitively probed in subpopulations of patients with specific characteristics using stratified analyses.

## Methods

### Data sources, search strategy, and selection criteria

This study was conducted and reported according to the meta-analysis of observational studies in epidemiology protocol [32]. Any observational study published in English and examining the relationship between EGFR status and OS in NSCLC patients with brain metastases was eligible for inclusion in our study, and no restrictions was placed on publication status (published, or in press). Briefly, we searched PubMed, EmBase, and Cochrane library for studies published up to September 2017 using the following search terms: (“brain metastases” OR “cerebral metastases” OR “neoplasm metastasis” OR “central nervous system” OR “encephalon”) AND (“epidermal growth factor receptor” OR “receptor, epidermal growth factor” OR “EGFR” OR “EGFR mutation”) AND (“lung” OR “pulmonary”) AND (“neoplasms” OR “cancer” OR “carcinoma” OR “neoplasm”) AND “human” AND “English”. We also conducted manual searches of reference lists from all relevant original and review articles to identify additional eligible studies. Study title, study design, disease status, exposure, control, and outcome variables were used to identify relevant studies. All analyses were based on previously published studies; thus, no ethical approval or patient consent were required.

The literature search and study selection were independently undertaken by 2 authors using a standardized approach, and any inconsistencies were settled by the primary author until a consensus was reached. Studies were included in this meta-analysis if they met the following criteria: (1) Study design: study with prospective or retrospective observational design; (2) Participants: adult NSCLC patients (≥18 years old) with brain metastases; (3) Exposure: EGFR mutations and wide-type; and (4) Outcomes: OS. Studies were excluded for the following reasons: (1) Participants with other disease; (2) patients with other histologies NSCLC; (3) No available data for statistics; or (4) Publication type was a review, comment, or letter to the editor.

### Data collection and quality assessment

The following data from each study were extracted independently by two authors: first author’s name, publication year, country, study design, disease stage, percentage of adenocarcinoma, sample size, mean age, percentage male, percentage smokers, percentage TKI, and OS. Any disagreements were resolved by a consensus. We also utilized a 9-star system using the Newcastle-Ottawa Scale (NOS) for assessing the quality of the observational studies based on selection (4 items), comparability (1 item), and outcome (3 items) [33]. We regarded a study with a score ≥ 7 as being of high quality.

### Statistical analysis

We examined the relationship between EGFR status and OS for NSCLC patients with brain metastases based upon hazard ratios (HRs) and 95% confidence intervals (CIs) published in each study. The random-effects model was employed to calculate summary HRs and 95% CIs for EGFR mutations versus EGFR wild-type for OS [34, 35]. Potential heterogeneity across the studies was examined using the Cochran’s Q-statistic and I^2^ statistic [36, 37]. A sensitivity analysis was conducted by removing each individual study from the meta-analysis [38]. Meta-regression was performed to explore the source of heterogeneity based on percentage of adenocarcinoma, sample size, mean age, percentage male, percentage smokers, and percentage TKI [39]. Subgroup analyses were conducted based on country (Eastern versus Western), study design (prospective versus retrospective), disease status (adenocarcinoma versus both), sample size (≥500 versus < 500), mean age (≥65.0 versus < 65.0 years), percentage male (≥50.0% versus < 50.0%), percentage smokers (≥50.0% versus < 50.0%), percentage of patients receiving TKIs (≥30.0% versus < 30.0%), and study quality (high versus low). The ratio between subgroups was calculated via the Chi-square test and meta-regression [39]. Publication bias was qualitatively evaluated using a funnel plot and quantitatively assessed using the Egger and Begg tests [40, 41]. All reported *P* values are 2-sided and *P* values < 0.05 are regarded as statistically significant for all included studies. Statistical analyses were conducting using STATA software (version 10.0; Stata Corporation, College Station, TX, USA).

## Results

### Literature search

**Fig. 1:**
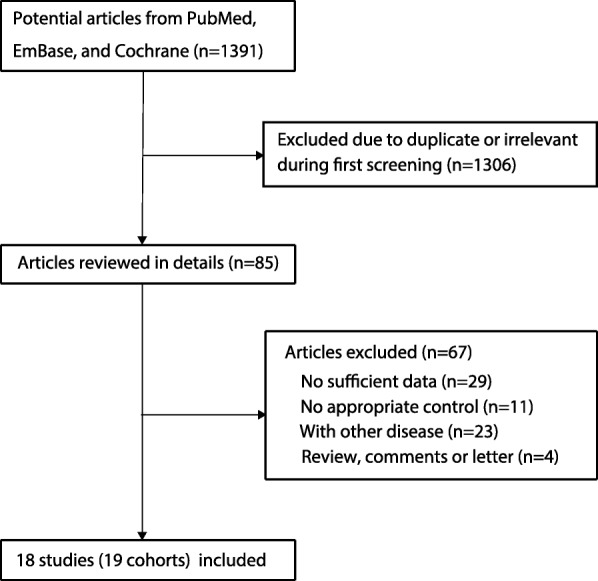
Flow diagram of the study selection process

A total of 591 citations from PubMed, 681 citations from EmBase, and 119 citations from the Cochrane Library were identified, for a total of 1391 records. Of these, 1306 were excluded as duplicates or irrelevant studies. Eighty-five studies were selected for full-text assessment, of which 67 studies were excluded: 29 studies lacked sufficient data, 11 studies lacked appripriate controls, 23 studies reported patients with other disease, and 4 studies were reviews, comments, or letters to the editor. Finally, 18 studies were pooled into the meta-analysis [14–31]. The study selection process is presented in Fig. 1.

### Characteristics of selected studies

Of the 18 included studies (encompassing a total of 4373 NSCLC patients with brain metastases), 3 had a prospective design [18, 26, 30] and 15 had a retrospective design [14–17, 19–25, 27–29, 31]. Nine included studies were from Asia [14, 15, 17, 18, 20, 24–26, 31], and 9 were from Europe or America [16, 19, 21–23, 27–30]. All studies were published between 2009 and 2017, and 41–1127 patients were included in each study. Five studies included only patients with lung adenocarcinoma [14, 18, 20, 22, 26], while the other studies included various types of lung cancers [15–17, 19, 21, 23–25, 27–31]. The mean age of included patients ranged from 55.0–67.0 years, percentage male ranged from 28.3 to 66.2%, percentage smokers ranged from 21.9 to 77.4%, and percentage of patients receiving TKI ranged from 5.1 to 44.0%. Study quality was assessed using the NOS, and 4 studies scored 8 [19, 20, 22, 23], 5 studies scored 7 [14–16, 25, 26], 5 studies scored 6 [21, 27, 28, 30, 31], and the remaining 4 studies scored 5 [17, 18, 24, 29]. The general characteristics of the included studies are presented in Table 1.

**Table 1:** Baseline characteristic of studies included in the systematic review and meta-analysis

Author	Publication year	Country	Study design	Disease stage	Percentage of adenocarcinoma (%)	Sample size	Mean age (years)	Percentage male (%)	Percentage of smoker (%)	Percentage of TKI (%)	NOS score
Hsiao [14]	2013	China	Retrospective	I–IV	100.0	139	NA	41.0	30.0	32.0	7
Han [20]	2016	China	Retrospective	I–IV	100.0	234	NA	53.4	44.0	30.3	8
Russo [21]	2017	Italy	Retrospective	IIIb-IV	73.0	137	66.2	59.1	77.4	NA	6
Baek [15]	2016	Korea	Retrospective	IV	70.0	259	68.0	64.5	59.1	NA	7
Stanic [22]	2014	Slovenia	Retrospective	I–IV	100.0	629	64.0	51.8	72.0	NA	8
Hsu [16]	2016	Canada	Retrospective	IV	NA	543	66.0	40.0	NA	41.0	7
Hendriks [23]	2014	The Netherlands	Retrospective	NA	91.9	124	61.9	41.9	72.6	37.1	8
Iuchi [17]	2014	Japan	Retrospective	Ia-IV	79.4	1127	67.0	65.2	64.2	42.3	5
Arrieta [30]	2009	Mexico	Prospective	IIIb-IV	64.8	293	60.7	56.0	53.9	12.6	6
Lee [24]	2012	China	Retrospective	I-III	93.0	43	59.0	53.0	37.0	44.0	5
Iuchi [18]	2013	Japan	Prospective	NA	100.0	41	NA	29.3	21.9	NA	5
Li [25]	2015	China	Retrospective	NA	83.8	136	NA	52.9	58.8	30.9	7
Luo [31]	2013	China	Retrospective	I-IV	82.4	136	55.0	61.0	37.5	5.1	6
Zhuang [26]	2013	China	Prospective	I-IV	100.0	54	61.7	42.6	NA	42.6	7
Tomasini [27]	2016	France	Retrospective	IV	89.4	142	62.0	66.2	76.1	NA	6
Eichler [19]	2010	US	Retrospective	I-IV	94.0	93	60.9	33.0	57.0	19.0	8
Mak [28]	2015	US	Retrospective	NA	NA	172	60.0	45.0	70.0	42.0	6
Wang [29]	2015	US	Retrospective	I-III	85.9	71	61.9	50.7	NA	21.1	5

### Meta-analysis

**Fig. 2:**
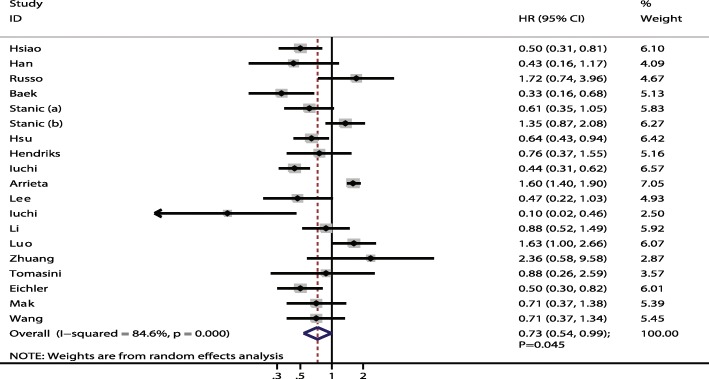
Forest plot of the relationship between EGFR mutation and OS in NSCLC patients with brain metastases. Each study is shown by the point estimate of the HR and 95% CI (extending lines)

**Fig. 3:**
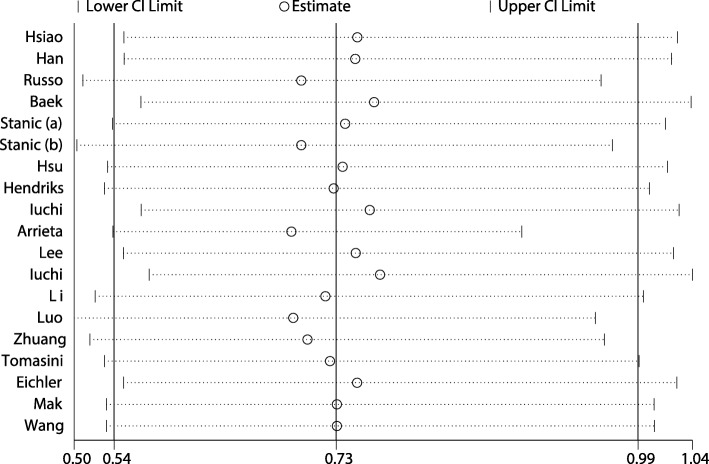
Results of the sensitivity analysis. Results when each study is excluded are shown by the point estimate of the HR and 95% CI (extending lines)

After pooling all included studies, a statistically significant improvement in OS was found for NSCLC patients with brain metastases with EGFR mutations compared with wild-type EGFR (HR: 0.73; 95% CI: 0.54–0.99; *P* = 0.045; Fig. 2). Substantial heterogeneity was detected across the included studies (I^2^ = 84.6%; *P* < 0.001), so we conducted a sensitivity analysis (Fig. 3 and Table 2). The results of the sensitivity analysis were consistent after excluding several studies [21, 22, 25–27, 31], with the magnitude of heterogeneity not significantly decreasing. However, when we excluded the study conducted by Arrieta et al. [30], the heterogeneity among the included studies decreased to 66.9% and EGFR mutations showed large improvement for OS (HR: 0.69; 95% CI: 0.54–0.89; *P* = 0.005). This study specifically included a lower percentage of adenocarcinoma, which may have contributed to lower incidence of EGFR mutations.

**Table 2:** Sensitivity analysis

Excluding study	HR and 95% CI	*P* value	Heterogeneity (%)	P value for heterogeneity
Hsiao [14]	0.75 (0.55–1.03)	0.073	84.3	< 0.001
Han [20]	0.75 (0.55–1.02)	0.069	85.1	< 0.001
Russo [21]	**0.70 (0.51–0.96)**	**0.027**	**85.2**	**< 0.001**
Baek [15]	0.76 (0.56–1.04)	0.087	84.2	< 0.001
Stanic (a) [22]	0.74 (0.54–1.02)	0.064	85.0	< 0.001
Stanic (b) [22]	**0.70 (0.51–0.97)**	**0.033**	**85.2**	**< 0.001**
Hsu [16]	0.74 (0.53–1.02)	0.065	84.7	< 0.001
Hendriks [23]	0.73 (0.53–1.00)	0.052	85.4	< 0.001
Iuchi [17]	0.76 (0.56–1.03)	0.076	81.8	< 0.001
Arrieta [30]	**0.69 (0.54–0.89)**	**0.005**	**66.9**	**< 0.001**
Lee [24]	0.75 (0.55–1.02)	0.070	85.0	< 0.001
Iuchi [18]	0.77 (0.57–1.04)	0.089	84.3	< 0.001
Li [25]	**0.72 (0.52–1.00)**	**0.049**	**85.4**	**< 0.001**
Luo [31]	**0.69 (0.50–0.96)**	**0.026**	**84.9**	**< 0.001**
Zhuang [26]	**0.71 (0.52–0.96)**	**0.029**	**85.3**	**< 0.001**
Tomasini [27]	**0.73 (0.53–0.99)**	**0.046**	**85.4**	**< 0.001**
Eichler [19]	0.75 (0.55–1.03)	0.073	84.4	< 0.001
Mak [28]	0.73 (0.53–1.01)	0.056	85.3	< 0.001
Wang [29]	0.73 (0.53–1.01)	0.056	85.3	< 0.001

### Meta-regression and subgroup analyses

The heterogeneity test showed a *P* < 0.05, indicating that heterogeneity was statistically significant in the overall analysis. Therefore, a meta-regression analysis was conducted based on percentage of adenocarcinoma, sample size, mean age, percentage male, percentage smokers, and percentage receiving TKI to evaluate potential sources of heterogeneity. The results of the analysis are presented in Additional file 1. Overall, we noted that the percentage of patients receiving TKI might affect the relationship between EGFR status and OS in NSCLC patients with brain metastases (*P* = 0.015), while percentage of adenocarcinoma (*P* = 0.279), sample size (*P* = 0.671), mean age (*P* = 0.112), percentage male (*P* = 0.275), and percentage smokers (*P* = 0.196) were all not significant factors contributing to this relationship.

The results of the subgroup analysis are listed in Table 3. Overall, we noted that EGFR mutations were associated with significantly improved OS in studies conducted in Eastern Countries (HR: 0.59; 95% CI: 0.37–0.92; *P* = 0.021) and in those with retrospective design (HR: 0.70; 95% CI: 0.55–0.89; *P* = 0.004), sample size ≥500 (HR: 0.52; 95% CI: 0.36–0.76; *P* = 0.001), mean age of included patients ≥65.0 years (HR: 0.59; 95% CI: 0.36–0.98; *P* = 0.043), percentage male < 50.0% (HR: 0.60; 95% CI: 0.43–0.82; *P* = 0.002), percentage receiving TKI ≥ 30.0% (HR: 0.60; 95% CI: 0.48–0.75; *P* < 0.001), and in studies with high quality (HR: 0.67; 95% CI: 0.50–0.90; *P* = 0.007). No other significant differences were detected based on other pre-defined factors. Furthermore, there was no evidence of a factor-specific difference in the HR for OS among patients with EGFR mutations compared to patients with wild-type EGFR (Table 3).

**Table 3:** Subgroup analyses

Group	Number of cohorts	HR and 95% CI	*P* value	Heterogeneity (%)	*P* value for heterogeneity	Ratio between subgroups	P value between- subgroup
Country							
Eastern Countries	9	0.59 (0.37–0.92)	0.021	76.3	< 0.001	0.68 (0.38–1.21)	0.189
Western Countries	10	0.87 (0.61–1.25)	0.455	81.6	< 0.001		
Study design							
Prospective	3	0.81 (0.18–3.75)	0.792	83.7	0.002	1.16 (0.25–5.38)	0.852
Retrospective	16	0.70 (0.55–0.89)	0.004	64.7	< 0.001		
Disease status							
Adenocarcinoma	6	0.65 (0.36–1.18)	0.154	75.5	0.001	0.86 (0.43–1.72)	0.660
Both	13	0.76 (0.53–1.10)	0.152	86.7	< 0.001		
Sample size							
500 or more	2	0.52 (0.36–0.76)	0.001	49.4	0.160	0.68 (0.41–1.10)	0.117
< 500	17	0.77 (0.56–1.06)	0.106	81.4	< 0.001		
Mean age (years)							
65.0 or older	4	0.59 (0.36–0.98)	0.043	73.2	0.011	0.65 (0.35–1.19)	0.160
< 65.0	11	0.91 (0.65–1.28)	0.595	78.0	< 0.001		
Percentage male (%)							
50.0 or greater	12	0.81 (0.55–1.19)	0.290	86.2	< 0.001	1.35 (0.82–2.23)	0.242
< 50.0	7	0.60 (0.43–0.82)	0.002	42.9	0.105		
Percentage of current/past smokers (%)							
50.0 or greater	11	0.78 (0.52–1.17)	0.239	87.5	< 0.001	1.50 (0.64–3.50)	0.349
< 50.0	5	0.52 (0.25–1.11)	0.092	81.1	< 0.001		
Percentage of patients using TKI (%)							
30.0 or greater	9	0.60 (0.48–0.75)	< 0.001	26.2	0.211	0.59 (0.32–1.10)	0.099
< 30.0	4	1.01 (0.57–1.81)	0.963	87.3	< 0.001		
Study quality							
High	10	0.67 (0.50–0.90)	0.007	58.0	0.011	0.85 (0.47–1.53)	0.585
Low	9	0.79 (0.47–1.31)	0.357	88.3	< 0.001		

*CI* confidence intervals, *HR* hazards ratios, *TKI* tyrosine kinase inhibitor, bold entries P<0.05

### Publication Bias

**Fig. 4:**
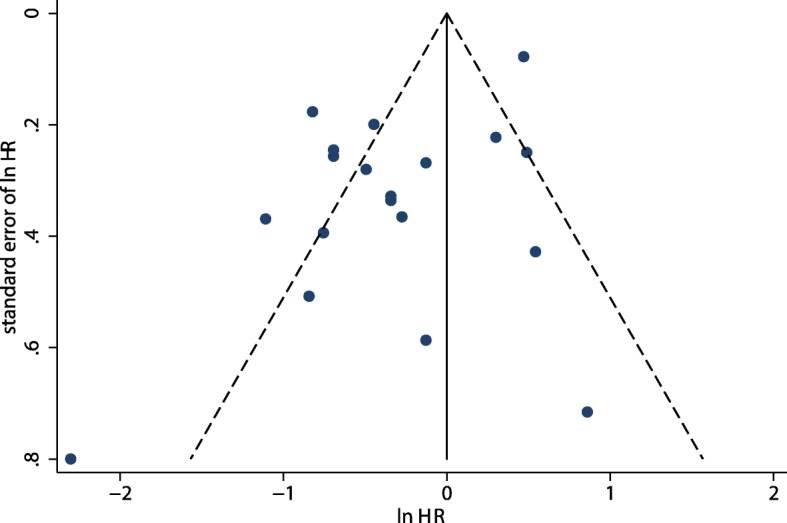
Publication bias

Potential publication bias was detected via funnel plot (Fig. 4). Although the Begg test [41] showed no evidence of publication bias (*P* = 0.889), the Egger test [40] showed potential evidence of publication bias (*P* = 0.008). The results were not changed following adjustment with the trim and fill method (HR: 0.73; 95% CI: 0.54–0.99; *P* = 0.045) [42].

## Discussion

Our meta-analysis analyzed 3 prospective studies and 15 retrospective studies to explore possible correlations between EGFR status and OS for NSCLC patients with brain metastases. The included 18 studies involved 4373 NSCLC patients with brain metastases. Our results support that mutated EGFR associates with significant improvement in OS compared with wild-type EGFR. Stratified analyses determined that a similar effect was seen in studies conducted in Eastern Countries and studies with retrospective design, sample size ≥500, mean age of included patients ≥65.0 years, percentage male < 50.0%, percentage receiving TKI ≥ 30.0%, and studies with high quality.

The results were predominantly consistent with those of previous studies, which have demonstrated that EGFR mutations could prolong OS over wild-type EGFR. Hsiao et al. [14] suggested that EGFR mutation is an independent predictive factor for treatment response and OS in lung adenocarcinoma patients with brain metastases. They point out that radiation could induce nuclear translocation of wild-type, augmenting repair of DNA double-strand breaks in lung adenocarcinoma cells [43]. Alternatively, irradiation could delay DNA repair and decrease clonogenic survival in NSCLC cells with mutated EGFR [44, 45]. Baek et al. indicated that EGFR mutation could increase incidence of brain metastases and improve OS in NSCLC patients with brain metastases. This significant improvement in OS was mainly observed if the brain is the first metastatic site [15]. Several other studies have demonstrated EGFR mutations to associate with an increased risk of brain metastases and prolonged survival after brain metastases in NSCLC patients [16–19]. A possible reason could be that EGFR-TKI could prevent brain metastases progression in NSCLC patients with activating EGFR mutations compared with those treated with conventional chemotherapy [46]. Further, different mechanisms and drug sensitivities between synchronous and metachronous brain metastases exist since metachronous brain metastases always accompany central nervous system symptoms while synchronous brain metastases are often asymptomatic.

Several studies included in our analysis reported inconsistent results. Numerous studies found that EGFR mutations could not predict survival or local control in NSCLC patients with brain metastases [20–29]. This lack of significant difference could be due to the studies being designed to evaluate the incidence of brain metastases or the treatment effects as primary endpoint. The sample size of these studies was smaller than expected, and broad 95% CIs were found, leading to no statistically significant differences being found. In addition, Arrieta et al. suggested that EGFR expression significantly associated with worse OS, possibly due to the low frequency of EGFR expression [30]. Luo et al. suggested that median OS was 24.5 and 15.0 months in EGFR wild-type and mutant groups, respectively [31]. The major reason for this could be that the use of EGFR-TKI therapy after diagnosis of brain metastases was associated with longer survival [47, 48].

Subgroup analyses suggested that EGFR mutations were associated with improved OS in several subsets. The combined higher incidence of EGFR mutation and sufficient statistical power indicated a relationship between EGFR status and OS in NSCLC patients with brain metastases. Further, high percentages of patients using EGFR-TKI produced better effect on OS. This relationship was not observed in numerous subsets, and these conclusions may be unreliable since smaller cohorts were included. Therefore, we presented our relative results and provided a synthetic and comprehensive review.

Although this is not the first meta-analysis to evaluate the relationship between EGFR status and OS in NSCLC patients with brain metastases, this study has several strengths over the previous meta-analysis [49]. The main purpose of previous meta-analysis was to investigate the risk of brain metastases in EGFR mutations or wild type, and secondary outcome was OS in NSCLC patients with brain metastases. They point out EGFR mutation patients was associated with an increased risk of brain metastases than those with wild type. Further, EGFR mutation prolonged OS in patients with brain metastases compared with wild-type EGFR. However, the result for NSCLC patients with brain metastases just based on 7 studies, and numerous studies were neglected. Furthermore, no stratified analyses were conducted based on confounding factors. The current quantitative meta-analysis included 18 studies involved 4373 NSCLC patients with brain metastases comprised a wide range of characteristics of patients and the results of stratified analyses according to country, study design, disease status, sample size, mean age, percentage male, percentage smokers, percentage of patients receiving TKIs, and study quality were calculated.

The limitations of this study are as follows: (1) recursive partitioning analysis, Karnofsky Performance scale, activity of systemic disease, type of treatment, and disease status may be associated with OS in NSCLC with brain metastases, while these data were not available in mostly included studies or unable to conduct stratified analysis; (2) several other molecular alterations in central nervous system response to TKI therapy were not reported in mostly studies; (3) most included studies had a retrospective design, so recall and selection biases might affect the relationship found between EGFR status and OS; (4) publication bias is an inevitable problem since this study is based on published articles, and ongoing or unpublished studies were not included in this meta-analysis; and (5) inherent limitations are found in any meta-analysis using pooled data when individual data are not available.

## Conclusions

The pooled results show that EGFR mutation is associated with a significant improvement in OS when compared with wild-type EGFR. Further, this relationship manifested in studies conducted in Eastern Countries and studies with retrospective design, sample size ≥500, mean age of included patients ≥65.0 years, percentage male < 50.0%, percentage receiving TKI ≥ 30.0%, and high quality. Further large-scale prospective studies should be conducted to verify this relationship and explore this relationship in specific patient subsets, especially for broader implications of improved OS in patients with EGFR mutant brain metastases.

## Additional file

Additional file 1: **Figure S1.** meta-regression based on percentage of adenocarcinoma (*P* = 0.279). **Figure S2.** meta-regression based on sample size (*P* = 0.671). **Figure S3.** meta-regression based on mean age (*P* = 0.112). **Figure S4.** meta-regression based on percentage male (*P* = 0.275). **Figure S5.** meta-regression based on percentage smokers (*P* = 0.196). **Figure S6.** meta-regression based on percentage of patients receiving TKI (*P* = 0.015). (DOCX 2864 kb)

## Abbreviations

CI: Confidence interval
EGFR: Epidermal growth factor receptor
HR: Hazard ratio
NOS: Newcastle-Ottawa Scale
NSCLC: Non-small-cell lung cancer
OS: Overall survival
TKI: Tyrosine kinase inhibitor
