# Supplementary material for: The role of EGFR mutation as a prognostic factor in survival after diagnosis of brain metastasis in non-small cell lung cancer: a systematic review and meta-analysis
Source: BMC Cancer. 2019 Feb 13;19:145. doi: 10.1186/s12885-019-5331-z (PMC6375157; doi:10.1186/s12885-019-5331-z)
Supplement: Supplementary file 1 — Figure S1. meta-regression based on percentage of adenocarcinoma (P = 0.279). Figure S2. meta-regression based on sample size (P = 0.671). Figure S3. meta-regression based on mean age (P = 0.112). Figure S4. meta-regression based on percentage male (P = 0.275). Figure S5. meta-regression based on percentage smokers (P = 0.196). Figure S6. meta-regression based on percentage of patients receiving TKI (P = 0.015). (DOCX 2864 kb) [file 12885_2019_5331_MOESM1_ESM.docx]

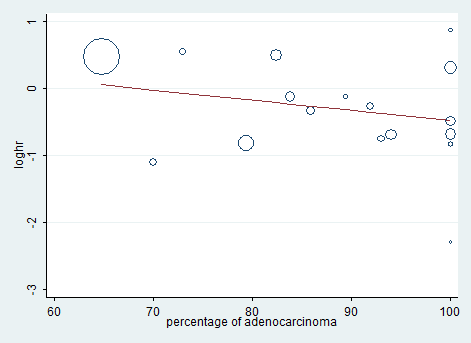


Figure S1. meta-regression based on percentage of adenocarcinoma (P=0.279)


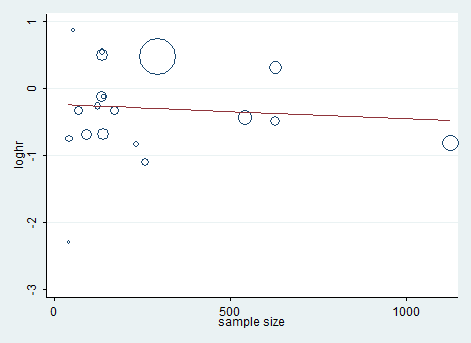


Figure S2. meta-regression based on sample size (P=0.671)


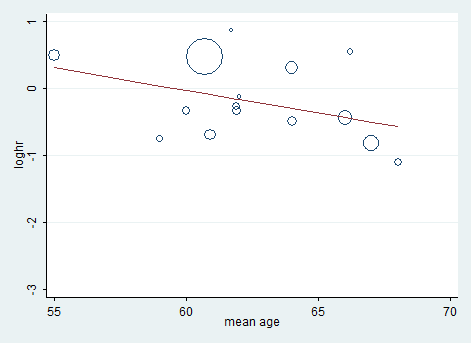


Figure S3. meta-regression based on mean age (P=0.112)


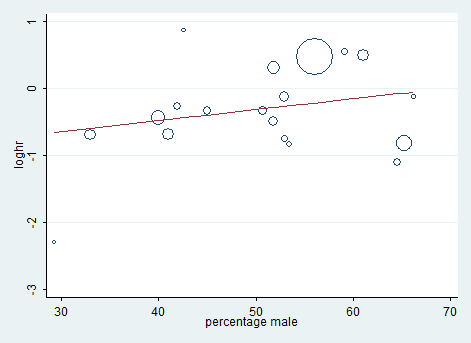


Figure S4. meta-regression based on percentage male (P=0.275)


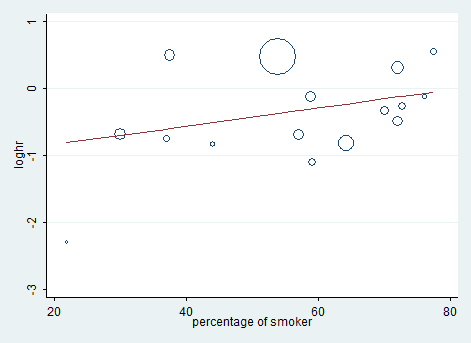


Figure S5. meta-regression based on percentage smokers (P=0.196)


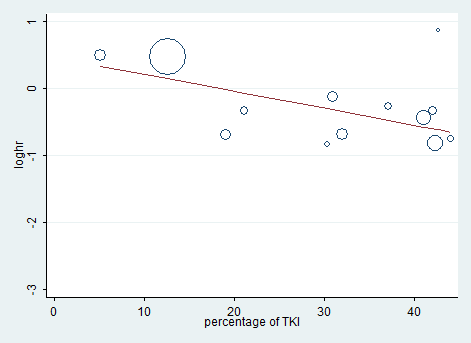


Figure S6. meta-regression based on percentage of patients receiving TKI (P=0.015)
